# Artificial intelligence in peritoneal dialysis: general overview

**DOI:** 10.1080/0886022X.2022.2064304

**Published:** 2022-04-26

**Authors:** Qiong Bai, Wen Tang

**Affiliations:** Department of Nephrology, Peking University Third Hospital, Beijing, China

**Keywords:** Artificial intelligence, peritoneal dialysis, machine learning

## Abstract

**Objective:**

This article is a general overview about artificial intelligence/machine learning (AI/ML) algorithms in the domain of peritoneal dialysis (PD).

**Methods:**

We searched studies that used AI/ML in PD, which were classified according to the type of algorithm and PD issue.

**Results:**

Studies were divided into (a) predialytic stratification, (b) peritoneal technique issues, (c) infections, and (d) complications prediction. Most of the studies were observational and majority of them were reported after 2010.

**Conclusions:**

There is a number of studies proved that AI/ML algorithms can predict better than conventional statistical method and even nephrologists. However, the soundness of AI/ML algorithms in PD still requires large databases and interpretation by clinical experts. In the future, we hope that AI will facilitate the management of PD patients, thus increasing the quality of life and survival.

## Introduction

The prevalence of end-stage renal disease (ESRD) continues to rise and it is a significant healthcare burden worldwide [1]. Peritoneal dialysis (PD) is a well-established renal replacement therapy (RRT) modality with clinical and economic advantages for ESRD patients [2]. The survival of patients treated with PD is equivalent to those who receive hemodialysis (HD) and PD had better quality of life than HD patients [3,4].

Artificial intelligence (AI) solutions are currently present in all medical and nonmedical fields. With extensive utilization of big data, AI is expanding its influences in healthcare and has gradually changed the way clinicians pursue for problem-solving [5]. Machine learning (ML) is a subset of AI that allows the computer to perform a specific task without explicit instructions. Instead of adopting a theory-driven strategy that requires a preformed hypothesis from prior knowledge, training an ML model typically follows a data-driven approach that allows the model to learn from experience alone. Specifically, the model improves its performance iteratively on a training set by comparing the predictions to the ground truths and adjusting model parameters so as to minimize the distance between the predictions and the truths. It has been demonstrated that ML solutions for a better prediction of events beat human accuracy [6–9].

AI/ML has recently been applied in many health-related realms, including medical imaging and diagnostics [10,11], drug discovery and development [12], treatment and prediction of diseases [8], and management of patient records and hospital administration [13]. Additionally, a few recent studies implemented AI methods in kidney disease and renal replacement treatment field. These models were developed to estimate the risk of short-term mortality following dialysis [14], calculate the future eGFR values [15], or choose an optimal dialysis prescription [16]. Nevertheless, the implementation of AI solutions in the dialysis field is still at the beginning. This review’s purpose is to summarize and depict the current research and impact of AI/ML algorithms on peritoneal dialysis (PD).

## Methods

We searched the electronic databases of PubMed and EMBASE from its earliest date until July 2021 for published articles using keywords: ‘artificial intelligence’, ‘machine learning’, ‘deep learning’, ‘data mining’, ‘dialysis’, and ‘peritoneal dialysis’. The reference sections of relevant articles were also searched manually for additional publications. The studies referring to AI in PD included randomized controlled trials (RCTs) and observational studies, reviews and meta-analyses. All trials were listed in Table 1. We summarized these studies and impact of AI on PD: how does it work, what are the potential benefits and how it can help in improving the healthcare in PD patients. Some AI/ML algorithms will be presented in a simplified way to help readers understanding how it works.

**Table 1.** AI studies involved in PD.

Study	Type of PD issues	Number of samples	Type of AI/ML algorithm	Outcome
Zhang 2005 [17]	Patients stratification	–	Fuzzy logic	Provide PD schemes
Chen 2006 [18]	Patients stratification	111 patients	Neural network	Stratify peritoneal membrane transporter
Tangri 2008 [19]	Technique issue	3269 patients	Neural network	Predict early PD technique failure
Tangri 2011 [20]	Technique issue	3269 patients	Neural network	Predict PD technique failure
Zhang 2017 [21]	Acute peritonitis	83 patients, 49 biomarkers	SVM, Neural network, RF	Define pathogen in PD patients with bacterial infections
Rodrigues 2017 [22]	Other complications	850 patients	Naïve Bayes, Multilayer Perceptron, k-NN, RF, Data mining	Predict stroke
Brito 2019 [23]	Other complications	2489 samples	Data mining	Classify the values of serum creatinine in patients undergoing CAPD procedures
Tang 2019 [24]	Other complications	656 patients	Neural network, GRU	Predict mortality
Wu 2020 [25]	Other complications	22859 patients	RF	Predict prolonged length of hospital stay
Noh 2020 [26]	Other complications	1730 patients	Neural network	Predict mortality
Kong 2021 [27]	Other complications	23992 patients	SVM, k-NN, RF	Predict prolonged length of hospital stay

SVM: support vector machine; RF: random forest; k-NN: k- nearest neighbor; GRU: gated recurrent unit; CAPD: continuous ambulatory peritoneal dialysis.

## AI/ML algorithm approach

**Figure 1.**
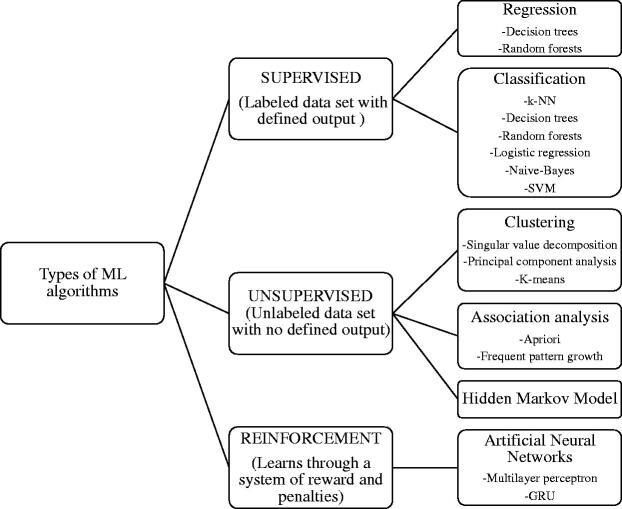
Types of ML algorithms. k-NN: k- nearest neighbor; SVM: support vector machine; GRU: gated recurrent unit.

Core concepts, various AI algorithms, and differences between them have been defined and described elsewhere [28–30]. ML algorithms (Figure 1) included Naive Bayes models, multilayer perceptron, support vector machine (SVM). k-nearest neighbor (k-NN), random forest (RF) and neural network algorithms were used. Two trials used data mining algorithms, and one had fuzzy logic approaches.

Naive Bayes algorithm works on Bayes theorem and takes a probabilistic approach. The algorithm has a set of prior probabilities for each class. Once data is fed, the algorithm updates these probabilities to form something known as posterior probability. This comes useful when you need to predict whether the input belongs to a given list of classes or not [31].

SVM is an algorithm that classifies data. It essentially filters data into categories, which is achieved by providing a set of training examples, each set marked as belonging to one or the other of the two categories. The algorithm then works to build a model that assigns new values to one category or the other.

K-NN algorithm uses a bunch of data points segregated into classes to predict the class of a new sample data point. It estimates how likely a data point is to be a member of one group or another. It essentially looks at the data points around a single data point to determine what group it is actually in.

A decision tree is a flow-chart-like tree structure that uses a branching method to illustrate every possible outcome of a decision. Each node within the tree represents a test on a specific variable – and each branch is the outcome of that test.

Random forest or ‘random decision forest’ is an ensemble learning method, combining multiple algorithms to generate better results for classification, regression and other tasks. Each individual classifier is weak, but when combined with others, can produce excellent results. The algorithm starts with a ‘decision tree’ (a tree-like graph or model of decisions) and an input is entered at the top. It then travels down the tree, with data being segmented into smaller and smaller sets, based on specific variables. Random forests offer a more accurate classifier as compared to Decision tree algorithm.

An artificial neural network (ANN) comprises ‘units’ arranged in a series of layers, each of which connects to layers on either side. ANNs are inspired by biological systems, such as the brain, and how they process information. ANNs are essentially a large number of interconnected processing elements, working in unison to solve specific problems. ANNs also learn by example and through experience, and they are extremely useful for modeling non-linear relationships in high-dimensional data or where the relationship amongst the input variables is difficult to understand. Compared to logistic regression, ANNs are more flexible, and thus more susceptible to overfitting. Network size can be restricted by decreasing the number of variables and hidden neurons, and by pruning the network after training [32].

## Clinical approach

Trials dealing with AI and PD covered four issues: (a) predialytic stratification, (b) peritoneal technique issues, (c) infections, and (d) complications and mortality prediction (Table 1). Most of the studies were observational and majority of them were reported after 2010.

### Patients stratification

Since high peritoneal membrane transport status is associated with higher morbidity and mortality, determining peritoneal membrane transport status can result in a better prognosis. A study used artificial neural network (ANN) model for predialytic stratification of 111 uremic patients on the basis of peritoneal membrane transport status from a 5-year PD database [18]. The evaluation of peritoneal membrane transport status by the ANN model, if predictable before PD, will help clinicians make decisions about more suitable dialysis modality. Another application of AI in PD was the selection of PD schemes. Fuzzy logic algorithm was used to provide offers about PD schemes which showed excellent compatibility with doctors’ opinions [17].

### Technique failure

PD technique failure remains an important and frequent complication of PD treatment and is associated with significant risk to patients and health services. The first year has been recognized as a particularly vulnerable period, with studies estimating that just less than one-half of patients who experience technique failure in the 1st year of therapy [33–36].

Early technique failure is a major impediment to the growth of PD as a treatment option globally.

Understanding risk factors for early technique failure can help nephrologists develop interventions that may mitigate it. A study used a large, high-quality and prospectively collected data from the United Kingdom Renal Registry [20] between 1999 and 2004, included 3269 patients and created ANN model to predict technique survival. Multilayer, ‘perceptron’, ANNs with 73-80-1 nodal architectures were constructed and trained using the backpropogation approach. PD center significantly impacts PD technique survival. Most physical examination characteristics, laboratory data and comorbid conditions do not confer a significant effect on the likelihood of technique failure for PD patients. In addition, ANN-based model performed reasonably well in predicting early technique failure among incident PD patients. The ANN performed significantly better than a traditional, LR-based prediction model [19].

### Acute peritonitis prediction

Peritonitis is a common complication of PD and remains a major cause of early dropout and mortality. However, although highly elevated white cell counts with a proportion of >50% granulocytes in the peritoneal effluent are used as indicators of peritonitis, culture-based diagnosis of infection is slow and unsatisfactory. Treatment of peritonitis therefore continues to be largely empirical. ML techniques were demonstrated to identify specific biomarker signatures associated with Gram-negative and Gram-positive organisms and with culture-negative episodes of unclear etiology. A study used a systematic approach to characterize responses to microbiologically well-defined infection in a total of 83 PD patients on the day of presentation with acute peritonitis. They applied different ML models, including SVM, NN, and RF, to complex biomedical datasets and identified key pathways involved in pathogen-specific immune responses at the site of infection [21]. It demonstrated the power of advanced mathematical models to analyze complex biomedical datasets and highlight critical pathways involved in pathogen-specific inflammatory responses at the site of infection and had diagnostic and prognostic implications by providing patient treatment choice.

### Complications and mortality prediction

AI/ML algorithms would help predict impending complications such as fluid overload, heart failure, or stroke, allowing early detection and interventions to avoid hospitalization and provide better healthcare to improve patients’ prognosis and reduce costs.

The hospital admission rate is high in PD patients. Accurate prediction of length of stay (LOS) can provide useful prognostic information that may help clinicians make optimal use of medical resources and produce better clinical decisions. A recent study developed a scoring tool for predicting prolonged length of stay (pLOS) in 22,859 PD patients by combining machine learning and traditional logistic regression (LR). Three machine learning methods, classification and regression tree (CART), RF, and gradient boosting decision tree (GBDT), were used to develop models to predict pLOS. The scoring system took advantage of the superior prediction performance of the machine learning model and the interpretability of the traditional LR model. The RF model had the best prediction performance among the three machine learning models in terms of overall prediction performance, discrimination, and calibration and thus was used to identify the 10 most predictive variables for building the scoring system [25]. In 2021, they developed the pLOS prediction model using a stacking model constructed with SVM, RF and k-NN algorithms and conducted validation. It was showed that the stacking model was superior in overall performance, discrimination, calibration, balanced accuracy, and accuracy [27].

Continuous ambulatory peritoneal dialysis (CAPD) patients need to be monitored using routine blood tests on follow-up. Applying data mining to the vast amounts of data collected from tests (e.g., consecutive creatinine values) to discover patterns becomes meaningful [23]. The classification process can find patterns useful to understand the patients’ health development. In addition, data mining and ML can take a simple and meaningless blood's test data set and build it into a Decision Support System, which can predict CAPD patients with a stroke risk according to their routine blood tests [22]. A study (including 850 cases) used five different AI algorithms, including Naïve Bayes, Logistics Regression (LR), MLP, Random Tree (RT), and k-NN, to predict the stroke risk of CAPD patients. RT and k-NN had the best results with the sensitivity, specificity and accuracy higher than 95% [22].

A Korean study assessed mortality risk prediction in 1,730 PD patients using ML algorithms. It was showed that deep neural network significantly outperformed logistic regression method. ML-based model could provide mortality prediction in Korean PD patients [26]. Another study used deep models, including recurrent neural network (RNN) and gated recurrent unit (GRU), to predict mortality in Chinese PD patients based on their routine clinical data. The recurrent neural network model, especially the GRU model, was demonstrated more effective in predicting PD patients’ prognosis as compared with the LR model [24].

In short, ML algorithms can benefit PD patients and nephrologists with high predictions (risk of stroke, infection, cardiovascular events [37], and even mortality risk) through easily accessible and large amounts of clinical data (demographic, biological, or PD-related data).

## Current challenges and future perspectives

Instead of adopting a theory-driven strategy that requires a preformed hypothesis from prior knowledge, training an ML model typically follows a data-driven approach that allows the model to learn from experience alone. AI/ML produces insights based on a data set, but the precise way in which it concludes/results may not be visible. Thus, people have difficulty of understanding it, which make people reluctant to use it [38]. Thus, future studies which focus on the interpretability of the AI results are needed. Some studies have already emerged in this field [39,40]. Secondly, another important limitation of the AI/ML approach is that there is a need for robust validation in real-world studies. ML has been applied to prediction of prognosis as a means of stratifying treatment. It should be noticed that models trained on different data might draw different conclusions, leading to descriptions of different populations. The extent to which predictors may actually represent proxies for severity that may be specific to a particular health system or setting. These circumstances do not necessarily undermine the usefulness of a model, but they should raise concern for generalizability [41]. Although it has been demonstrated that AI/ML algorithms outperform traditional statistical method, there is still a long way to improve clinical practice. More studies with clinical evaluation and validation are needed. Moreover, as use of AI requires both knowledge and experience, it is also worth noting that all models have the so-called hyperparameters that require clinical experts estimate. As a result, the soundness of AI/ML algorithms in healthcare requires large databases, long periods of ‘training’ and interpretation by clinical experts. At last, in the future, AI/ML devices will predict dialysis complications through simple clinical variables, which even could monitor the entire dialysis process. Using AI/ML solutions to mine knowledge from big data registries will allow building intelligent systems (the so-called Clinical Decision Support Systems), which will help clinicians in classifying risks, diagnosing PD complications, assessing prognosis and thus improve the healthcare of PD patients.

## Conclusions

There is a number of studies proved that AI/ML algorithms can predict better than conventional statistical method and even nephrologists. AI/ML algorithms are implemented in predialytic patient stratification, PD technique issue, peritonitis, cardiovascular complication, stroke and mortality prediction, thus minimizing mortality and admission rate. However, interpretability of the AI study needs to be established in the future study to increase their potential utilization.
